# miR-202-5p Inhibits Lipid Metabolism and Steroidogenesis of Goose Hierarchical Granulosa Cells by Targeting ACSL3

**DOI:** 10.3390/ani13030325

**Published:** 2023-01-17

**Authors:** Mingxia Ran, Shenqiang Hu, Qingyuan Ouyang, Hengli Xie, Xi Zhang, Yueyue Lin, Xuejian Li, Jiwei Hu, Liang Li, Hua He, Hehe Liu, Jiwen Wang

**Affiliations:** Farm Animal Genetic Resources Exploration and Innovation Key Laboratory of Sichuan Province, Sichuan Agricultural University, Chengdu 611130, China

**Keywords:** miR-202-5p, ACSL3, lipid, steroid hormone, goose granulosa cell

## Abstract

**Simple Summary:**

Poultry laying performance depends on the normal development of ovarian follicles. Lipid metabolism and steroidogenesis in granulosa cells are essential for maintaining normal follicular development; therefore, a detailed understanding of the molecular mechanisms underlying granulosa cell functions may provide a basis for improving production. In the present study, we investigated the effects of miR-202-5p, which show expression changes during follicular development, on lipid metabolism and steroidogenesis in goose hierarchical follicular granulosa cells. We found that miR-202-5p significantly inhibited lipid deposition and steroid hormone production in goose hierarchical follicles granulosa cells via *ACSL3*. These results revealed the important role of miR-202-5p in the regulation of goose granulosa cell functions and demonstrate that this miRNA is a potential target for molecular breeding.

**Abstract:**

miRNAs are critical for steroidogenesis in granulosa cells (GCs) during ovarian follicular development. We have previously shown that miR-202-5p displays a stage-dependent expression pattern in GCs from goose follicles of different sizes, suggesting that this miRNA could be involved in the regulation of the functions of goose GCs; therefore, in this study, the effects of miR-202-5p on lipid metabolism and steroidogenesis in goose hierarchical follicular GCs (hGCs), as well as its mechanisms of action, were evaluated. Oil Red O staining and analyses of intracellular cholesterol and triglyceride contents showed that the overexpression of miR-202-5p significantly inhibited lipid deposition in hGCs; additionally, miR-202-5p significantly inhibited progesterone secretion in hGCs. A bioinformatics analysis and luciferase reporter assay indicated that Acyl-CoA synthetase long-chain family member 3 (*ACSL3*), which activates long-chain fatty acids for the synthesis of cellular lipids, is a potential target of miR-202-5p. ACSL3 silencing inhibited lipid deposition and estrogen secretion in hGCs. These data suggest that miR-202-5p exerts inhibitory effects on lipid deposition and steroidogenesis in goose hGCs by targeting the *ACSL3* gene.

## 1. Introduction

The egg-laying performance of poultry is mainly determined by the growth and development of ovarian follicles, which are related to the differentiation of granulosa cells (GCs) and oocyte maturation [1,2,3]. During follicular growth, maturation, and ovulation, GCs transport follicular nutrients, provide mechanical support, and synthesize steroids [4,5]; additionally, investigations of lipid profiles in both follicular cells (including cumulus, granulosa, and theca cells) and follicular fluid by mass spectrometry suggest that lipid metabolism in GCs is also pivotal for follicular development and oocyte maturation [6,7,8]. In birds, lipid metabolism in GCs may be particularly crucial given the large amounts of liver-synthesized yolk precursors (mainly lipids) [9,10]. De novo lipogenesis has been observed in GCs. These synthesized fatty acids may be transported to oocytes through gap junctions, membrane fusion, or binding to oocyte membrane receptors and participate in oocyte maturation, lipid deposition, the maintenance of membrane integrity and fluidity, and other regulatory processes during the normal development of follicles [11,12]. De novo lipogenesis has also been detected in goose GCs [13,14]. Lipid metabolism in GCs is also closely related to the synthesis and secretion of steroid hormones. Steroidogenesis requires a constant supply of cholesterol as a precursor for conversion to steroids. There are multiple sources of cholesterol for steroidogenesis, including de novo synthesis within the ER and delivery from circulating low-density lipoprotein and high-density lipoprotein [15,16]; it is, therefore, of great importance to clarify the mechanisms underlying lipid metabolism in GCs. 

microRNAs (miRNAs) are endogenous RNAs of approximately 23 nucleotides. They function via interactions with protein-coding genes to direct their posttranscriptional repression by imperfectly binding to the 3′ untranslated regions (3′UTRs) [16,17]. miRNAs play significant regulatory roles in various organisms, including roles in development and host–pathogen interactions as well as cell differentiation, proliferation, apoptosis, and tumorigenesis [18]. Microarray chips, high-throughput sequencing, northern blotting, and other technical means have recently been used to confirm that miRNAs exist in almost all vertebrate ovarian tissues. They are the most abundant small RNA molecules in the ovary. After the knockout of the Dicer gene (encoding the key enzyme for miRNA cleavage and maturation) in mouse GCs, the number of ovarian primordial follicles decreased sharply and the number of atretic follicles increased [19], confirming that miRNAs are necessary to maintain the normal growth and development of follicles; furthermore, it has also been reported to regulate steroid hormone synthesis and lipid metabolism [16,20,21,22]. MiR-202-5p expression is much higher in the granular layer than in the membrane layer, according to our previous study, and its expression pattern increases initially and then decreases in the 4–6 mm, 8–10 mm, and F5 granular layers [23,24]. These results suggest that miR-202-5p is an important regulator during goose follicle development.

In the present study, the effects of miR-202-5p on lipid metabolism and steroid hormone secretion in GCs were investigated by lipid droplet staining and the detection of intracellular cholesterol, triglyceride, extracellular estradiol, and progesterone levels. More importantly, we determined the downstream target gene of miR-202-5p and its effects on lipid deposition and steroid hormone secretion in GCs. These data provide insights into the role of miR-202-5p in lipid metabolism and steroidogenesis in goose GCs. 

## 2. Materials and Methods

### 2.1. Animals

The healthy maternal line of Tianfu meat geese (*Anser cygnoides*), laying at least two and three eggs regularly, was used. The Waterfowl Breeding Experimental Farm at Sichuan Agricultural University (Chengdu, China) provided food and water to the geese under natural light and temperature conditions. The laying cycles of each goose were recorded, and ovarian follicles were collected from all geese in the same laying cycle. All selected geese were euthanized by the inhalation of carbon dioxide and cervical dislocation, performed by competent, experienced personnel who applied the technique correctly. Based on diameters, the ovarian follicles were divided into two classes: pre-hierarchical (6 to 10 mm in diameter) and hierarchical (F5–F1, F1 > F2 > F3 > F4 > F5 in diameter) follicles. 

### 2.2. Granulosa Cell Culture

According to a previous study, the granulosa layer of each follicle is separated from the theca layer [25]. The granulosa layers separated from F2 to F4 follicles were washed with PBS (pH 7.4) and dispersed in 0.05% type II collagenase (Sigma, St. Louis, MO, USA). The cells were diluted to 6 × 10^5^/mL by Dulbecco’s Modified Eagle’s Medium/Nutrient Mixture (F12, containing 3% fetal bovine serum; Sigma) and then cultured in 12-well or 96-well culture plates in a humidified atmosphere of 5% CO_2_ and 95% air at 37 °C. 

### 2.3. Cell Transfection

Hierarchical granulosa cells (hGCs, F2-F4) were cultured in fresh medium before RNA transfection. Mimic (UUUCCUAUGCAUAUACUUAUUUU), mimic-NC (UUGUACUACACAAAAGUACUG), inhibitor (AAAGAAGUAUAUGCAUAGGAAA), inhibitor-NC (CAGUACUUUUGUGUAGUACAA), and siRNAs (Table 1) were transfected into cells using Lipofectamine 3000 (ThermoFisher Scientific, Carlsbad, CA, USA, according to the manufacturer’s instructions. Cells were harvested for RNA extraction 24 h post-transfection.

### 2.4. RNA Extraction and qRT-PCR Analysis

After total RNA was extracted by TRIzol reagent (Invitrogen, Carlsbad, CA, USA), the quality, purity, and concentration of RNA were determined by spectrophotometry; after that, PrimeScript RT kit (TaKaRa, Dalian, China) was used to synthesize cDNA from total RNA once the RNA quality met the requirements for subsequent use, according to the manufacturer’s instructions; then, qRT-PCR was performed using 2 SYBR Premix Ex Taq II (TaKaRa). As stated in the introduction, for 0.5 μL of each gene-specific primer (10 μM), we prepared 12 μL of reaction solution by mixing 1 μL cDNA with 6.25 μL SYBR Ex Taq and 4.25 μL ddH_2_O [26]. Transcript levels of each sample were normalized to *GAPDH* using the 2^ΔΔ Ct^ method. The primers used in qRT-PCR are summarized in Table 2.

### 2.5. Oil Red O Staining and Detection of Intracellular Lipids 

The difference in lipid droplet deposition after 48 h of transfection with mimic/inhibitor/siACSL3 was detected by using Oil Red O staining. According to the previously described protocol, following three washes with PBS, the cells were fixed with 4% formaldehyde for 30 min at room temperature; afterward, dye was used with 0.3% filtered Oil Red O for 1 h at room temperature, and washed with 60% isopropanol for 10 s to remove free Oil Red O; then we added a suitable amount of PBS, and took photos under the microscope (Olympus, Tokyo, Japan). After removing the PBS and extracting the Oil Red O for 20 min with isopropanol, the supernatant was transferred to 96-well culture plates; finally, absorbance was measured at 510 nm using a spectrophotometer [14]. 

### 2.6. Measurement of Intracellular Triglyceride and Cholesterol Concentrations

Total protein isolated by radioimmunoprecipitation assay (RIPA) buffer (Thermo Fisher Scientific, Waltham, MA, USA) and phenylmethanesulfonyl fluoride (PMSF) was collected to determine the concentrations of intracellular triglycerides (TG) and total cholesterol (CH) by using the Goose TG and CH ELISA Assay Kits (Nanjing Jiancheng Bioengineering Institute, Nanjing, China). Results were evaluated by the colorimetric method [27]. 

### 2.7. Determination of Progesterone and Estradiol Production 

The production of Progesterone (P_4_) and Estradiol (E_2_) in the supernatant culture medium was detected using the Goose P4 and E2 ELISA Kit (Huding Biotechnology, Shanghai, China), according to the manufacturer’s instructions. 

### 2.8. Prediction of MiR-202-5p Target Genes

Target genes of gga-miR-202-5p (5′-UUUCCUAUGCAUAUACUUCUUU-3′) were analyzed for sites complementary to the miR-202-5p seed sequence by using both miRDB (http://mirdb.org/, accessed on 19 January 2022) and TargetScan (https://www.targetscan.org/vert_80/, accessed on 19 January 2022). Genes predicted by both miRDB and TargetScan were considered potential target genes of miR-202-5p.

### 2.9. Dual Luciferase Assays

The dual luciferase assays were used to confirm the binding site of miR-202-5p. According to the previous procedure, the plasmid (wild-type or mutant pmiRGLO-3′UTR-ASCL3) and miR-202-5p mimic or mimic negative control were co-transfected into HEK293T cells in 48-well plates using Lipofectamine 3000. After 48 hours of transfection, the activity of the luciferase was measured by Dual-Luciferase Reporter Assay Kit (Beyotime Biotechnology, Haimen, China) on a fluorescence/multi-detection microplate reader (US BioTek Laboratories, Shoreline, WA, USA) [28]. Three different experiments’ data are presented as means and standard deviations.

### 2.10. Statistical Analysis

All experimental data were analyzed by ANOVA or nonparametric tests, according to the results of homogeneity of variance tests. IBM SPSS Statistic (version 20, Chicago, IL, USA) was used for all statistical analyses. Results are presented as the mean ± SEM. The differences were considered significant at *p* < 0.05.

## 3. Results

### 3.1. Identification and Characterization of the Goose miR-202-5p Precursor Sequence

**Figure 1:**
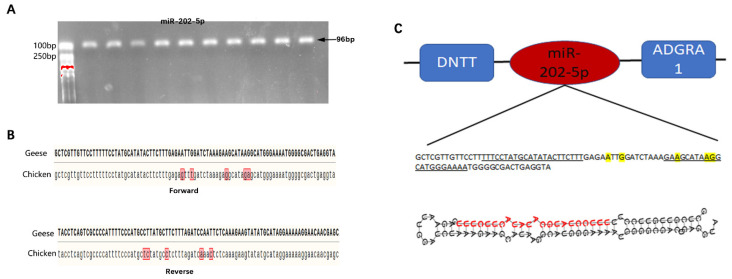
Sequence characteristics of the goose miR-202-5p precursor. (**A**) goose miR-202-5p precursor, (**B**) comparation of the miR-202-5p precursor between goose and chicken, and (**C**) genomic alignment and secondary structure prediction of goose miR-202-5p precursor.

The mature miR-202-5p sequence obtained by miRNA sequencing was consistent with the mature miR-202-5p sequence in chicken; therefore, primers for amplifying the goose miRNA-202-5p precursor sequence were designed based on the chicken miRNA-202-5p precursor sequence. After PCR amplification and cloning, the target fragment met the expected length and was successfully cloned (Figure 1A). Further forward and reverse sequencing results showed that the goose miR-202-5p sequence contained 96 bases, with 5 base mutations compared with the chicken precursor sequence (Figure 1B); however, it contained a complete mature miRNA-202-5p sequence, identical to the mature miR-202-5p sequence of chicken. The precursor sequence was further compared to that of the Pink-footed goose (*Anser brachyrhynchus*), showing that miR-202-5 was located between the *DNTT* and *ADGRA* genes, consistent with the position of miR-202-5p in chickens, mice, humans, pigs, and other taxa (Figure 1C).

### 3.2. miR-202-5p Suppressed Lipid Deposition and Steroidogenesis of Goose hGCs 

**Figure 2:**
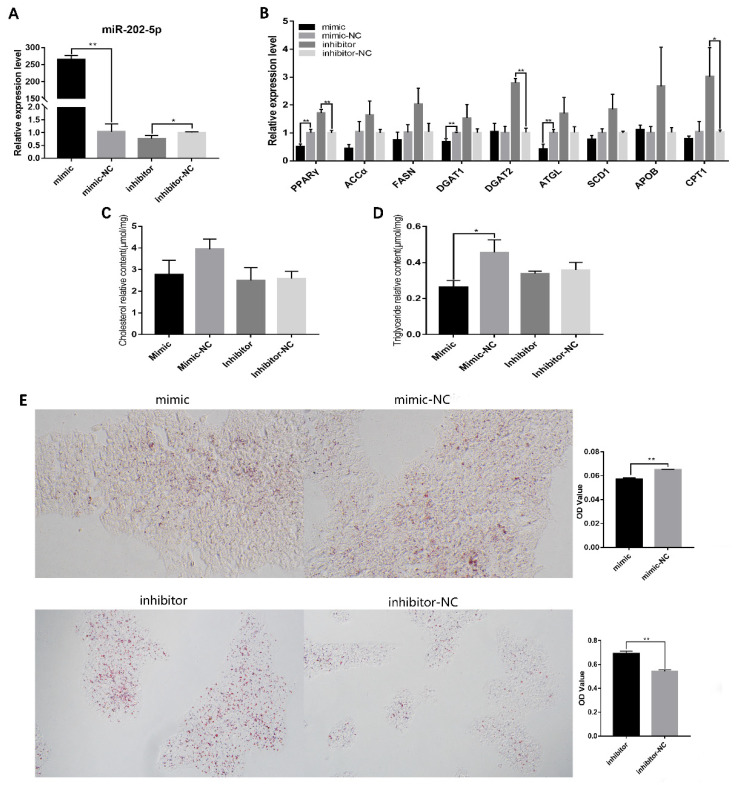
MiR-202-5p inhibits lipid deposition of goose granulosa cells. (**A**) effects of the mimic and inhibitor on expression of miR-202-5p, (**B**) effects of the interference or overexpression of miR-202-5p on the expression of genes related to lipid metabolism, (**C**,**D**) effect of the interference or overexpression of miR-202-5p on the content of TG and total CH (TG: triglyceride, CH: cholesterol), and (**E**) effect of the interference or overexpression of miR-202-5p on lipid deposition in hGCs. * *p* < 0.05, ** *p* < 0.01.

To further investigate the biological functions of miR-202-5p in hGCs, the miR-202-5p mimic and inhibitor were used to control the expression of miR-202-5p (Figure 2A). qRT-PCR showed that miR-202-5p significantly increased the relative expression levels of peroxisome proliferation activated receptor alpha (*PPARγ*) and diacylglycerol acyltransferase 1 (*DGAT1*), which promote lipid synthesis. The knock-down of miR-202-5p significantly elevated the mRNA levels of *PPARγ* and *DGAT2* (Figure 2B). This indicated that miR-202-5p may inhibit hGC lipid synthesis; therefore, we quantified the contents of intracellular CH and TG by ELISA and found that the overexpression of miR-202-5p significantly reduced the TG content in hGCs (*p* < 0.05, Figure 2B). Similarly, Oil Red O staining showed that the miR-202-5p mimic significantly decreased lipid deposition (*p* < 0.01), and the lipid deposition level increased significantly after miR-202-5p inhibitor transfection (*p* < 0.01, Figure 2C). These results indicated that miR-202-5p can inhibit lipid deposition in hGCs.

**Figure 3:**
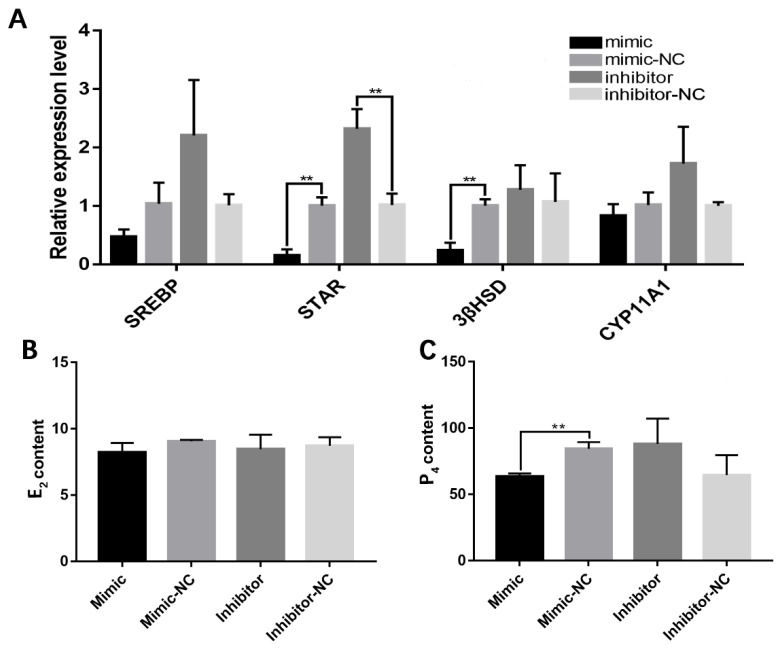
miR-202-5p blocks the secretion of steroid hormone in goose granulosa cells. (**A**) effects of the overexpression or interference of miR-202-5p on the expression levels of several steroidogenesis-related genes, and (**B**,**C**) effects of the overexpression or interference of miR-202-5p on the secretion of E2 and P4. ** *p* < 0.01.

A qRT-PCR analysis also showed that the miR-202-5p mimic significantly inhibited the expression of the steroidogenic acute regulatory protein (*StAR*) and 3β-hydroxysteroid dehydrogenase (*3βHSD*) (Figure 3A), suggesting that it has a regulatory role in steroid hormone synthesis. The effects of transfection with the miR-202-5p mimic or inhibitor on P_4_ and E_2_ secretion were also determined. The overexpression of endogenous miR-202-5p led to a significant decrease in P_4_ production (*p* < 0.01, Figure 3B,C). These data revealed that miR-202-5p inhibits steroid hormone secretion in hGCs.

### 3.3. miR-202-5p Inhibits Lipid Deposition and Steroidogenesis by Targeting ACSL3

**Figure 4:**
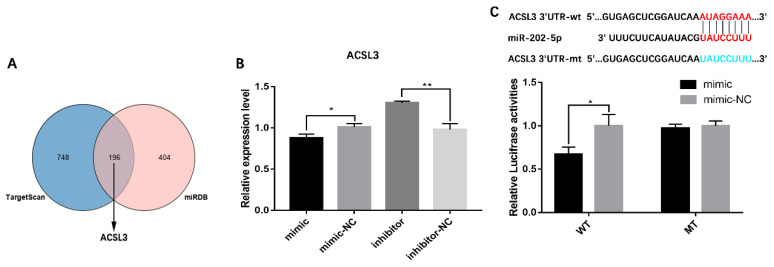
Identification of the targeting relationship between miR-202-5p and ACSL3. (**A**) predicted target genes from TargetScan and miRDB, and identification of candidate miR-202-5p target genes, (**B**) effect of miR-202-5p on the expression level of ACSL3 in GCs, and (**C**) experimental validation of the binding site of miR-202-5p in the 3′-UTR of ACSL3 by a luciferase reporter gene assay. wt, wild-type; mt, mutant-type. * *p* < 0.05, ** *p* < 0.01.

To ascertain the mechanism by which miR-202-5p inhibits hGC lipid synthesis, functional target genes of miR-202-5p were predicted using the miRDB and TargetScan databases. Among the predicted target genes of miR-202-5p, long-chain acyl CoA synthetase 3 (*ACSL3*) catalyzes the conversion of free long-chain fatty acids to acyl CoA esters (Figure 4A) [29]. This suggested that miR-202-5p may regulate lipid synthesis in hGCs by targeting *ACSL3*. qRT-PCR showed that the miR-202-5p mimic significantly inhibited the expression of *ACSL3* (*p* < 0.05), while the inhibitor significantly increased its expression (*p* < 0.01, Figure 4B); furthermore, to validate the targeted binding sites between miR-202-5p and *ACSL3*, a luciferase reporter assay was performed. As shown in Figure 4C, the luciferase activity of cells co-transfected with ACSL3-wt (wild type) and the miR-202-5p mimic was significantly lower than that of the miR-202-5p mimic-NC group, with no effect on the luciferase activity of cells transfected with the mutant-type binding site (*p* < 0.05). These results suggested that *ACSL3* is a downstream target gene of miR-202-5p.

**Figure 5:**
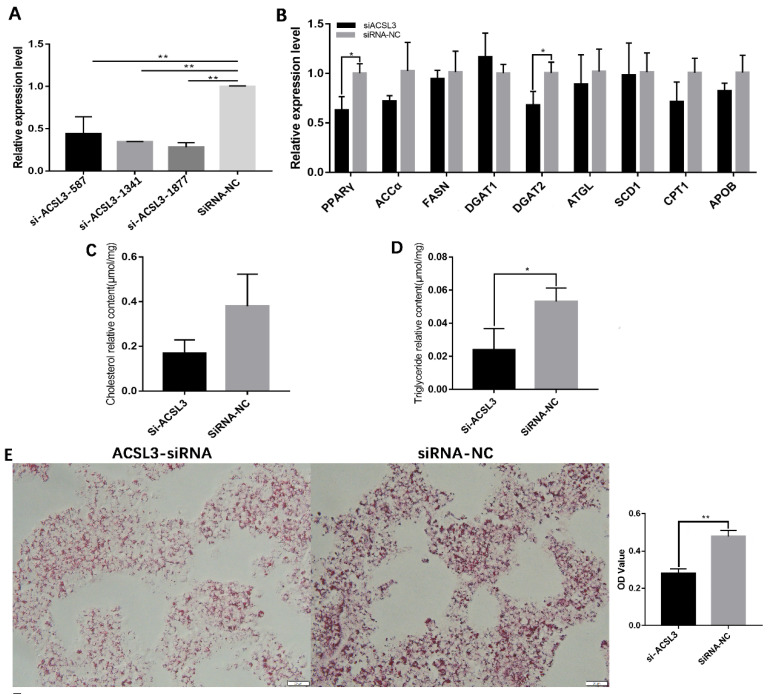
Downregulation of *ACSL3* inhibits lipid deposition by goose GCs. (**A**) effect of *ACSL3* siRNA on *ACLS3* mRNA expression, (**B**) effects of interference of *ACSL3* on the expression of genes related to lipid metabolism, (**C**,**D**) effect of si-ACSL3 on total TG and CH contents, and (**E**) effect of si-ACSL3 on lipid deposition in hGCs. * *p* < 0.05, ** *p* < 0.01.

The functions of *ACSL3* in hGCs were investigated using three ACSL3-siRNAs (namely, si-ACSL3-587, si-ACSL3-1341, and si-ACSL3-1877) targeting different sites in the coding region. As displayed in Figure 5A, the decrease in *ACSL3* mRNA expression was greatest for si-ACSL3-1877 (*p* < 0.01, Figure 5A); accordingly, si-ACSL3-1877 was selected for the following experiments; subsequently, the effects of si-ACSL3-1877 on lipid metabolism-related gene expression were evaluated by qRT-PCR. After *ACSL3* mRNA expression was knocked down by si-ACSL3-1877, the mRNA expression levels of *PPARγ* and *DGAT2* decreased significantly (*p* < 0.05, Figure 5B), indicating that *ACSL3* may be involved in the regulation of lipid synthesis in goose hGCs. *ACSL3* interference also significantly decreased the level of TG in hGCs. Oil Red O staining showed that lipid deposition levels were also significantly reduced in hGCs with ACSL3 interference (Figure 5C). These findings revealed that *ACSL3* enhances lipid synthesis in hGCs.

**Figure 6:**
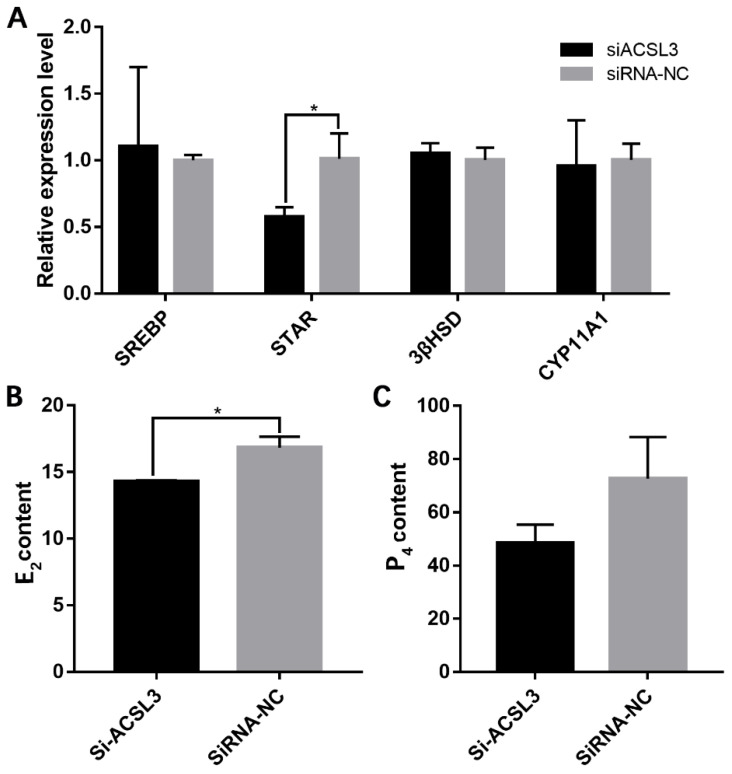
*ACSL3* inhibition decreased steroid hormone synthesis. (**A**) effects of si-ACSL3-1877 on the expression levels of several steroidogenesis-related genes, and (**B**,**C**) effects of *ACSL3* inhibition on the secretion of E2 and P4. * *p* < 0.05.

A qRT-PCR analysis of steroidogenesis-related genes showed that si-ACSL3-1877 significantly reduced *StAR* mRNA expression (Figure 6A); furthermore, analyses of extracellular E2 and P4 levels by ELISA revealed that the knock-down of *ACSL3* also significantly inhibited the secretion of E2 (Figure 6B). These data indicate that *ACSL3* promotes steroid hormone synthesis in hGCs. 

## 4. Discussion

While chickens, ducks, and various other birds lay about 300 eggs a year, the highest annual egg production of geese is only about 100. Most geese lay 20–40 eggs a year, which is a bottleneck restricting the development of the goose industry. The key molecular event that determines the annual egg production of poultry is selective dominance during follicular development, which was closely related to lipid and steroid hormone synthesis of granulosa cells; therefore, understanding the molecular mechanism regulating lipid and steroid hormone synthesis of granulosa cells can not only provide a theoretical basis for understanding the physiological mechanism of poor egg-laying performance of geese but also have important theoretical and practical significance for improving the egg production of geese. MiR-202-5p was first identified in the human testis, where it plays a key role in spermatogenesis. In ruminants, miR-202 shows restricted expression in bovine ovaries, with elevated expression in large healthy follicles, particularly in GCs [30]; furthermore, miR-202-5p levels are positively correlated with *CYP19A1* expression levels in goat ovarian follicles [31], indicating that miR-202-5p may play a critical role in GCs during follicle development. Lipid metabolism of GCs has a bi-directional effect on folliculogenesis and oocyte maturation. Increased levels of some lipids are a protective factor for folliculogenesis due to the requirement for fatty acids in meiotic resumption and fertilization in oocytes [32]. Our results showed that miR-202-5p significantly inhibited the expression of *PPARγ*, *DGAT1*, and *DGAT2* in hGCs. *PPARγ* is important in lipid metabolism and it regulates genes involved in the release, transport, and storage of fatty acids, such as lipoprotein lipase and the fatty acid transporter *CD36* [33]. Diacylglycerol acyltransferase 1 (DGAT1) and DGAT2 both catalyze the final, committed step of TG synthesis (the acylation of diacylglycerol with a fatty acyl-CoA) [34]; furthermore, the biogenesis of starvation-induced lipid droplets requires *DGAT1* [35]. *DGAT2* can also relocalize around lipid droplets, where it appears to be required for lipid droplet-localized TG synthesis [36]. This indicated that miR-202-5p may participate in lipogenesis of hGCs by inhibiting the expression of *PPARγ*, *DGAT1*, and *DGAT2*; furthermore, as determined by Oil Red O staining, miR-202-5p had a significant inhibitory effect on lipid deposition in goose hGCs; furthermore, miR-202-5p significantly decreased the level of intracellular TG. Follicular GCs are one of the main sites of ovarian steroid synthesis [37]. Steroidogenesis is a complex process by which cholesterol is transported to mitochondria and is converted via a series of enzymatic steps to steroid hormones [38,39]; therefore, lipid metabolism in GCs is closely related to the synthesis of steroid hormones. Our results showed that the overexpression of miR-202-5p could significantly inhibit the expression of steroidogenic acute regulatory protein (*StAR*). *StAR* is responsible for mediating the rate-limiting step in the movement of cholesterol from the outer to the inner mitochondrial membrane for steroidogenesis [38,40]. More importantly, we detected the effect of miR-202-5p on P4 and E2 synthesis in hGCs. The overexpression of miR-202-5p significantly inhibited the synthesis of P4. Collectively, these results suggested that miR-202-5p inhibits lipid deposition and P4 synthesis in hGCs.

To further reveal the molecular mechanism by which miR-202-5p inhibits lipid deposition in goose GCs, its potential target genes were predicted using the miRDB [41] and TargetScan [42] databases. Among the predicted target genes, *ACSL3* has been reported to be involved in regulating the conversion of free long-chain fatty acids (MUFAs) to acyl CoA esters [25], implying that *ACSL3* also participates in the regulation of lipid synthesis in GCs. The qRT-PCR analysis showed that miR-202-5p could inhibit the expression of *ACSL3*. A dual-luciferase assay further revealed that miR-202-5p can inhibit the function of *ACSL3* by binding to its 3′-UTR. ACSL3-mediated acyl CoA esters exert a variety of cellular functions, including the regulation of energy and lipid metabolism and signal transduction [29]. It is distributed at the site of lipid droplet formation in the endoplasmic reticulum and participates in lipid droplet synthesis [43,44], and it affects the expression of steroid hormone synthesis-related genes in prostate cancer cells [45]. In the present study, we found that when *ACSL3* expression was knocked down, levels of both lipid droplets and intracellular TG were significantly reduced in goose hGCs; furthermore, *ACSL3* interference significantly decreased the E_2_ levels. *ACSL3* was a target gene of miR-202-5p and promoted lipid deposition and E_2_ secretion in goose hGCs. 

## 5. Conclusions

In conclusion, we found that miR-202-5p can significantly inhibit lipid deposition and estradiol production in goose-hierarchical follicular GCs; furthermore, we demonstrated that miR-202-5p could directly target *ACSL3* by binding to its 3’UTR seed sequence. *ACSL3* can promote the deposition of lipid droplets, increase intracellular triglyceride levels, and increase the production of progesterone. These results suggest that miR-202-5p inhibits lipid and steroid hormone synthesis of hGCs by targeting *ACSL3*. In addition to clarifying the functions of miR-202-5p in follicular development, we expect these results to guide molecular breeding aimed at improving egg production in geese.

## Figures and Tables

**Table 1:** ACSL3 siRNAs.

	Sense (5′–3′)	Anti-Sense (5′–3′)
**si-ACSL3-587**	GAGGUGACCACCAUCAUUATT	UAAUGAUGGUGGUCACCUCTT
**si-ACSL3-1341**	CAGCAACCCAGCGAUUUAUTT	AUAAAUCGCUGGGUUGCUGTT
**si-ACSL3-1877**	GCUCGAAAGAAAGGAUUUATT	UAAAUCCUUUCUUUCGAGCTT

**Table 2:** Primers for qRT-PCR.

Genes	Primers (5′–3′)		Tm (°C)	Size (bp)
GAPDH	F: TTTCCCCACAGCCTTAGCA	R: GCCATCACAGCCACACAGA	60	90
PPARγ	F: CCTCCTTCCCCACCCTATT	R: CTTGTCCCCACACACACGA	59	108
ACCα	F: TGCCTCCGAGAACCCTAA	R: AAGACCACTGCCACTCCA	56.6	163
FASN	F: TGGGAGTAACACTGATGGC	R: TCCAGGCTTGATACCACA	57	109
DGAT1	F: CCTGAGGAACTTGGACACG	R: CAGGGACTGGTGGAACTCG	59	265
DGAT2	F: CGCCATCATCATCGTGGT	R: CGTGCCGTAGAGCCAGTTT	60	113
CPT1	F: GTCTCCAAGGCTCCGACAA	R: GAAGACCCGAATGAAAGTA	56	193
ATGL	F: TCGCAACCTCTACCGCCTCT	R: TCCGCACAAGCCTCCATAAGA	60	300
APOB	F: CTCAAGCCAACGAAGAAG	R: AAGCAAGTCAAGGCAAAA	56	153
SCD1	F: GCCATCGGTCCTACAAAGC	R: AGCCAATGTGGGAGAAGAAA	60	180
SREBP	F: CGAGTACATCCGCTTCCTGC	R: TGAGGGACTTGCTCTTCTGC	60	92
STAR	F: AGAATCTTGACCTCTTTGACGCTG	R: GAGACGGTGGTGGATAACGGA	60	87
3βHSD	F: GACCTGGGGTTTGGAATTGAG	R: TAGGAGAAGGTGAATGGGGTGT	60	170
CYP11A1	F: AGGGAGAAGTTGGGTGTCTACGA	R: CGTAGGGCTTGTTGCGGTAGT	60	89

## Data Availability

Data will be available upon request to the corresponding author.
